# Double-blind placebo-controlled randomized clinical trial to assess the efficacy of montelukast in mild to moderate respiratory symptoms of patients with long COVID: E-SPERANZA COVID Project study protocol

**DOI:** 10.1186/s13063-021-05951-w

**Published:** 2022-01-06

**Authors:** Francisco Mera-Cordero, Sara Bonet-Monne, Jesús Almeda-Ortega, Ana García-Sangenís, Oriol Cunillera-Puèrtolas, Sara Contreras-Martos, Gemma Alvarez-Muñoz, Ramon Monfà, Marina Balanzo-Joué, Rosa Morros, Betlem Salvador-Gonzalez

**Affiliations:** 1 Primary Care EAP El Pla Sant Feliu de Llobregat, Primary Care Management Costa de Ponent, Catalan Institute of Health, L’Hospitalet de Llobregat, Spain; 2 Institut Universitari de Recerca en Atenció Primària Jordi Gol i Gurina (IDIAPJGol), Barcelona, Spain; 3 Pharmacy Unit SAP Baix Llobregat Centre, Primary Care Management Costa Ponent, Catalan Institute of Health, Cornellà de Llobregat, Spain; 4 Primary Care Research Support Unit (USR) Costa de Ponent, Primary Care Management Costa de Ponent, Catalan Institute of Health, Cornellà de Llobregat, Spain; 5 Clinical Trials Research Unit (UICEC) IDIAPJGol – Platform SCReN, Barcelona, Spain; 6 Primary Care Research Support Unit (USR) Costa Ponent, Institut Universitari de Recerca en Atenció Primària Jordi Gol i Gurina (IDIAPJGol), Cornellà de Llobregat, Spain; 7 Primary Care Covid Notification and Monitoring Unit UNSC Metropolitana SUD, Primary Care Management Costa de Ponent, Catalan Institute of Health, L’Hospitalet de Llobregat, Spain; 8 Department of Pharmacology and Therapeutics, Universitat Autònoma de Barcelona, Bellaterra, Spain

**Keywords:** Montelukast, Dyspnea, Randomized controlled trial, COVID-19, SARS-CoV-2, Long COVID, Primary care, Quality of life, Health status

## Abstract

**Background:**

The coronavirus disease 2019 (COVID-19) pandemic continues to affect the globe. After 18 months of the SARS-CoV-2 emergence, clinicians have clearly defined a subgroup of patients with lasting, disabling symptoms. While big strides have been made in understanding the acute phase of SARS-CoV-2 infection, the pathophysiology of long COVID is still largely unknown, and evidence-based, effective treatments for this condition remain unavailable.

**Objectives:**

To evaluate the efficacy of 10 mg oral montelukast every 24 h versus placebo in improving quality of life associated with mild to moderate respiratory symptoms in patients with long COVID as measured with the COPD Assessment Test (CAT) questionnaire. The secondary objectives will evaluate the effect of montelukast versus placebo on improving exercise capacity, COVID-19 symptoms (asthenia, headache, mental confusion or brain fog, ageusia, and anosmia), oxygen desaturation during exertion, functional status, and mortality.

**Methods and analysis:**

Phase III, randomized, double-blind clinical trial. We will include 18- to 80-year-old patients with SARS-CoV-2 infection and mild to moderate respiratory symptoms lasting more than 4 weeks. Participants will be randomly allocated in a 1:1 ratio to the intervention (experimental treatment with 10 mg/day montelukast) or the control group (placebo group), during a 28-day treatment. Follow-up will finish 56 days after the start of treatment. The primary outcome will be health-related quality of life associated with respiratory symptoms according to the COPD Assessment Test 4 weeks after starting the treatment. The following are the secondary outcomes: (a) exercise capacity and oxygen saturation (1-min sit-to-stand test); (b) Post-COVID-19 Functional Status Scale; (c) other symptoms: asthenia, headache, mental confusion (brain fog), ageusia, and anosmia (Likert scale); (d) use of healthcare resources; (e) mortality; (f) sick leave duration in days; and (g) side effects of montelukast.

**Ethics and dissemination:**

This study has been approved by the Clinical Research Ethics Committee of the IDIAPJGol (reference number 21/091-C). The trial results will be published in open access, peer-reviewed journals and explained in webinars to increase awareness and understanding about long COVID among primary health professionals.

**Trial registration:**

ClinicalTrials.govNCT04695704. Registered on January 5, 2021. EudraCT number 2021-000605-24. Prospectively registered.

## Strengths and limitations of this study

Montelukast is an authorized medicine with extensive experience of use, good tolerance, and a known safety profile at the dose used in this trial.

Currently (August 2021), several studies are evaluating the efficacy of montelukast in the acute phase of COVID-19, and two clinical trials evaluate montelukast efficacy in reducing SARS-CoV-2 hospital admissions.

In a previous empirical treatment with montelukast in a case series of patients with long COVID, clinical improvement of symptoms was observed.

Since long COVID is an emergent condition, there are no validated scales for symptoms and quality of life.

To overcome memory bias in participants, telephone calls are added between office visits.

## Background and rationale

From June 22, 2020, to June 30, 2021, 3,547,032 cases of coronavirus disease (COVID-19) have been reported in Spain, with 7.3% patients admitted to hospital, 0.7% admitted to intensive care units, and a mortality of 1.4% [1].

In patients with mild to moderate symptoms and severe to critical coronavirus disease, full recovery might take up to 2 and 3–6 weeks from the onset of symptoms, respectively [2].

A few months into the pandemic, it was observed that in some patients, symptoms persisted for more than 4 weeks. The prolongation of the disease is now known as long COVID [3–7]. Some studies estimate that long COVID affects 10% of patients with COVID-19 [8]. The probability of developing long COVID does not seem to be related to the severity of the acute phase or to some of the risk factors associated with poor prognosis (male sex, older age, and comorbidities) [6].

Current data suggest that patients with long COVID are primarily women (78.9%), between 30 and 59 years old (86.9%), and only 8.43% have been previously admitted to the hospital for SARS-CoV-2 infection. In 65% of patients with long COVID, symptoms persist at least for 6 months from the onset of the disease [9].

Long COVID is a multi-organ disease characterized by a wide range of symptoms, including cough, headache, arthralgia, fever, abdominal pain, asthenia, brain fog, and skin manifestations. Dyspnea is one of the most frequent symptoms, as well as difficulty in performing activities of daily living, including self-care and social activities [9–11].

Although much progress has been achieved in understanding the acute phase of SARS-CoV-2 infection, the physiopathology of long COVID is less known [5, 7]. It has not been yet elucidated whether chronic symptoms are directly caused by the viral infection in multiple organs, indirectly through hyperactivation of the immune system, or due to the development of autoimmunity [7].

### Physiopathology of SARS-CoV-2 infection

SARS-CoV-2 penetrates the human cell using angiotensin-converting enzyme 2 (ACE2) as a receptor. ACE2 is mainly expressed in the lung, but also in the heart, kidney, intestine, vascular endothelium, and others [12]. The inflammatory process produced at the pulmonary and extrapulmonary levels and the overall immune response have been identified as important mechanisms in the physiopathology of COVID-19 [13].

The inflammatory process is a common response to viral infections; however, SARS-CoV-2 can overactivate the immune system leading to a cytokine storm, very likely associated with disease progression and multiple organ failure [13].

The high prevalence of antinuclear antibodies and other autoimmune markers observed in patients with COVID-19 points at the potential usefulness of specific treatments [14]. Leukotrienes, pro-inflammatory metabolites that participate in the regulation of the immune response, are possibly involved in the respiratory symptoms derived from the systemic inflammation in long COVID [15–17].

Leukotriene antagonists (LTRAs) are a group of drugs used to treat symptoms of asthma and allergic rhinitis. Their bronchodilator action and inhibition of airway inflammation improve respiratory function and airway hyperresponsiveness. By decreasing the action of leukotrienes C4, D4, and E4 when binding to CysLT1 receptors in the lungs and bronchi, LTRAs diminish bronchoconstriction and inflammation [18–20].

Recent studies have proposed the use of montelukast in the acute phase of COVID-19 due to a possible antiviral and anti-inflammatory effect [21–23]. Montelukast could prevent the entry of SARS-CoV-2 into the cell because of its affinity with the ACE2 receptor, thus shortening the course and severity of the disease [22]. Moreover, it has been hypothesized that montelukast could reduce the replication cycle of the virus [23, 24].

Clinical experience in patients admitted for COVID-19 suggests that montelukast could be associated with a reduction in clinical deterioration [25]. There are currently two registered clinical trials that evaluate the efficacy of montelukast in acute SARS-CoV-2 infection in outpatients [26, 27]. The first trial will compare the efficacy of montelukast versus placebo in reducing emergency visits and hospital admissions [26]. The second trial will evaluate the effect of montelukast and favicovir in reducing hospital admissions [27]

A pilot study was carried out using montelukast off-label in patients with COVID-19 [28]. Dyspnea, chest pain, malaise, dry cough, and nasal symptoms improved, and patients could return to work sooner.

This trial is based on prior clinical results and the hypothesis that antileukotrienes can reduce the cytokine cascade of inflammation triggered by SARS-CoV-2 infection. The E-SPERANZA COVID clinical trial aims to demonstrate the efficacy of montelukast in reducing respiratory symptoms and improving the quality of life of patients with long COVID (> 4 weeks).

## Objectives

The main objective of this study is to evaluate the efficacy of 4 weeks of treatment with 10 mg/day of oral montelukast versus placebo in improving the health-related quality of life associated with mild to moderate respiratory symptoms in patients with long COVID as measured by the CAT questionnaire [29].

One of the secondary objectives is to evaluate the effect of montelukast versus placebo on improving the following: exercise capacity, COVID-19 symptoms (such as asthenia, headache, brain fog, ageusia, and anosmia), oxygen desaturation during exertion, functional status, mortality, use of healthcare resources, days of sick leave, and medication side effects. Additionally, we will evaluate antinuclear antibodies as markers of the response to montelukast.

## Methods and analysis

### Trial design

The trial design is a phase III, randomized, double-blind, placebo-controlled clinical trial of superiority, with a two-arm parallel-group design, in which patients will be randomized to study treatment or placebo (1:1 allocation ratio).

### Study setting

The study will be carried out in thirteen primary healthcare centers in four health areas of Catalonia and Aragon, Spain. The list of study sites can be found at the Spanish Clinical Studies Registry website (https://reec.aemps.es).

### Study period

The study will be carried out from August 1, 2021, to March 1, 2023.

### Participants

Subjects are aged 18 to 80 years diagnosed with SARS-CoV-2 infection and persistent mild to moderate respiratory symptoms lasting between 4 and 12 weeks since the onset of the disease. Subjects will be included in the study if they meet all the eligibility criteria shown in Table 1.

**Table 1:** Inclusion and exclusion criteria for the study participants

**Inclusion criteria: participants are required to meet all inclusion criteria.**	
a) Individuals ≥ 18 and ≤ 80 years old with SARS-CoV-2 infection (positive SARS-CoV-2 detection test (RT-PCR), antigenic test or equivalent < 10 days from the onset of symptoms) treated in primary care.	
b) Persistent respiratory symptoms lasting for more than 4 weeks and less than 1 year.	
c) Mild to moderate dyspnea: score from 1 to 3 at the beginning of the study according to the Modified Medical Research Council (mMRC) scale.	
d) Patient must sign an informed consent form.	
**Exclusion criteria: participants meeting one or more of the following criteria will be excluded.**	
a) Severity criteria: fever> 38 °C, O_2_ saturation < 93%.	
b) Patients with pneumonia in the acute/subacute phase due to SARS-Cov-2.	
c) Patients who have required hospital admission for SARS-Cov-2.	
d) Chronic respiratory disease: chronic obstructive pulmonary disease (COPD), asthma, bronchiectasis, pulmonary fibrosis, obstructive sleep apnea syndrome (OSAS), chronic respiratory failure from any cause, and home oxygen therapy.	
e) Use of montelukast or zafirlukast ≤ 30 days prior inclusion.	
f) Use of gemfibrozil.	
g) Hypersensitivity to montelukast, lactose intolerance, or any of the excipients of study treatment or placebo.	
h) Active malignancy and current or recent chemotherapy treatment (< 6 months).	
i) Medical history of human immunodeficiency virus (HIV) infection or any severe immunosuppression.	
j) Patients who have been in a clinical trial 30 days prior to the study.	
k) Pregnancy or planning a pregnancy.	
l) Breastfeeding mother.	
m) The principal investigator considers that the subject will not be able to perform the test procedures.	

The centers and investigators of the study were chosen from the network of collaborating centers with research experience that have agreed to participate.

### Outcomes

The primary outcome is health-related quality of life associated with respiratory symptoms according to the CAT Scale [29] 4 weeks after starting the treatment. CAT is a validated scale to quantify and monitor the impact of COPD on well-being and daily life. It consists of 8 questions (rated from 0 to 5 points) and a total score of 0–40 (0–9 mild, 10–20 moderate, 21–30 severe, and 31–40 very severe). A difference of 2 or more points in health status is considered clinically significant [30].

The following are the secondary outcomes:

- Exercise capacity will be measured as the number of repetitions performed in the 1-min sit-to-stand test (1MSTS) [31], which consists of sitting down and getting up from a chair with no hand support as many times as possible for 1 min while connected to a saturation monitor. Measurements include the number of repetitions and oxygen saturation before and after performing the exercise.
- The Post-COVID-19 Functional Status Scale (PCFS) [32] focuses on relevant aspects of daily life during post-infection follow-up. The scale is intended to help health professionals become aware of functional limitations and to determine the degree of disability.
- Severity of common symptoms of patients with long COVID will be evaluated using Likert scales [33] [0 (less severe) to 10 (most severe)]: asthenia, headache, mental confusion (brain fog), ageusia, and anosmia.
- Use of health services, counting number of visits: virtual, primary care center, emergency room, and hospital admissions.
- Mortality.
- Sick leave days at day 28 (end-of-treatment).
- Side effects of the drug occurring at any time of the study period will be assessed and recorded in the data collection platform eCRF.
- Treatment compliance (percentage of pills taken at the end of treatment) will be estimated at the end of treatment by counting the remaining pills in the bottle or, if not possible, by directly asking the patient.

### Sample size

In the data published by Wang et al. [34] to evaluate an intervention in stable COPD patients, a weighted common standard deviation of the CAT score of 5.69 was observed. Basing the sample calculation on these data, in a study of the superiority of montelukast compared to placebo with an intention-to-treat analysis, accepting an alpha risk of 0.05 and a beta risk under 0.2 in a two-tailed contrast, 142 individuals in the treatment group and 142 in the control group are needed to detect a difference of 2 units. We assume a 10% loss to follow-up rate.

### Recruitment

Potential participants will be identified by the collaborator investigators at primary health centers and contacted in office visits or by telephone. If participants with persistent respiratory symptoms score from 1 to 3 (mild to moderate) dyspnea according to the modified Medical Research Council scale (mMRC) [35] and they agree to participate, an appointment will be scheduled with a physician investigator to assess the eligibility and to sign the informed consent. The recruitment period is estimated to finish by May 2022 or when a sufficient sample size is reached.

### Randomization, allocation, implementation, and blinding process

Participants will be randomly allocated to the intervention (treatment) or control (placebo) group. The randomization will be carried out by a IDIAPJGol statistician not involved in the recruitment using the R statistical software to obtain computer-generated random numbers without stratification, in a 1:1 allocation ratio by blocks of 4. The distribution will be made by blocks to ensure the proportionality of treatments between centers.

The randomization list will be provided to the pharmacy, which will label the trial treatments accordingly. Each treatment will be identified with a unique code. The centers will have medication in sequential order. The medication code dispensed to the patient will be registered on the medication dispensing form and on the data collection logbook. Since this is a double-blind study, neither healthcare professionals, patients nor the research team will be aware of the allocated group. Montelukast pills and their placebo equivalent will be visually identical.

Unblinding of a participant’s treatment will be allowed when information of the treatment received is needed for the effective and secure management of the patient.

### Intervention

The intervention consists of a 28-day treatment, with oral administration of 1 capsule per day (research drug or placebo). Participants belonging to the:

- Intervention group will be treated with montelukast 10 mg
- Control group will receive the placebo (microcrystalline cellulose)

The Pharmacy Department of Bellvitge University Hospital will encapsulate montelukast and the placebo.

**Fig. 1:**
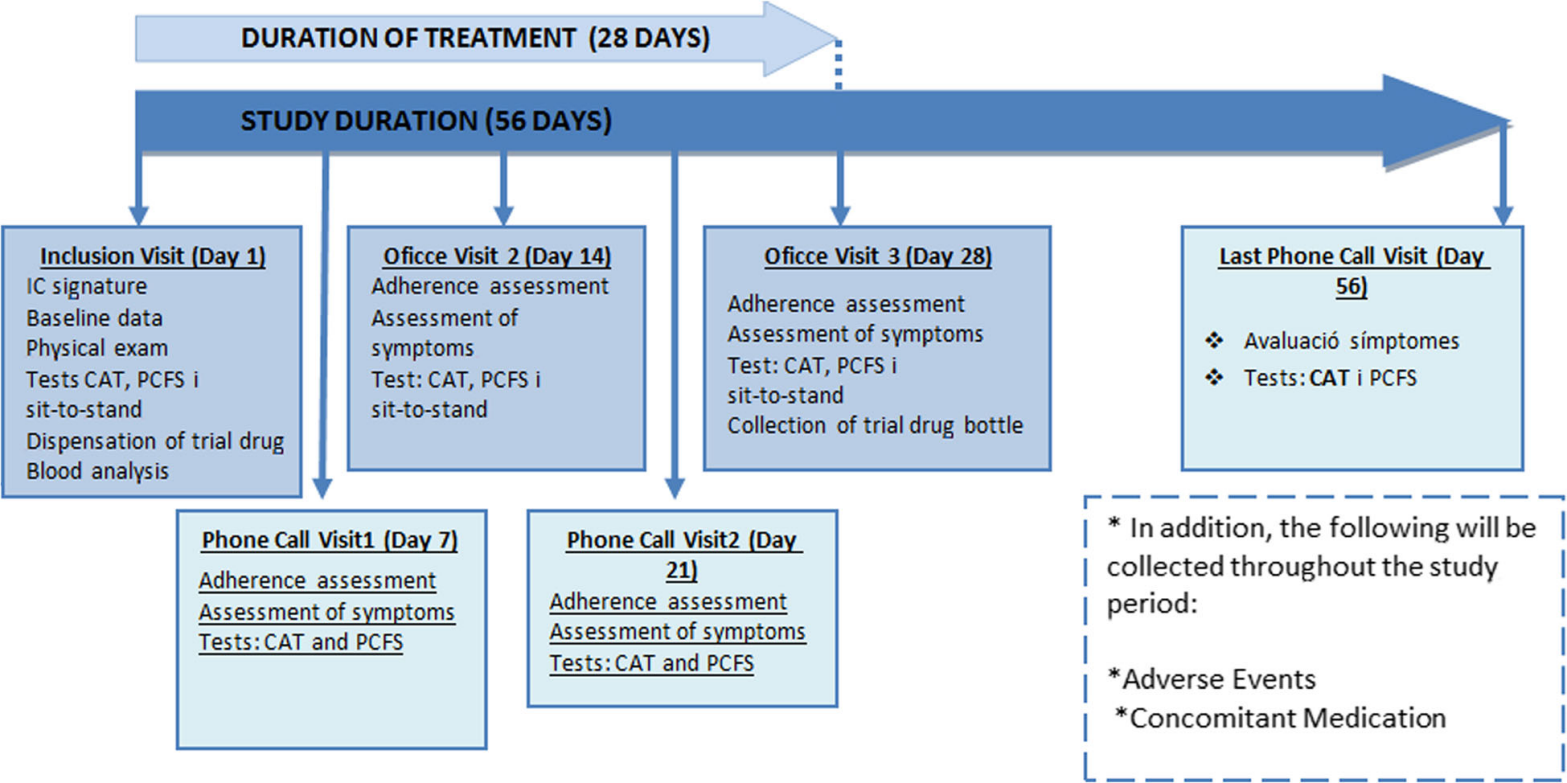
Summary and steps for the E-COVID study. IC, informed consent; CAT, COPD Assessment Test; PCFS, Post-COVID-19 Functional Status Scale; 1MSTS, 1-min sit-to-stand test

Follow-up consists of a series of office visits (days 1, 14, and 28) and telephone visits (days 7, 21 and 56), as described in Fig. 1 and Table 2. A window period of ± 2 days for each visit will be accepted.

**Table 2:** Time schedule of recruitment, enrollment, intervention, assessments, and visits

	Study period							
	Enrollment	Allocation	Post-allocation					Close-out
Time point**	***t***_***0***_	***0***	***t***_***1***_	***t***_2_*	***t***_***3***_	***t***_4_*	***t***_***5***_	***t***_f_*
**Enrollment**								
**Eligibility screen**	X	X						
**Informed consent**		X						
**Allocation**		X						
**Interventions**								
**Intervention [montelukast]**								
**Control [placebo]**								
**Assessments**								
**CAT scale**	X		X	X	X	X	X	X
**PCF Scale**			X	X	X	X	X	X
**1MSTS test**			X		X		X	
**Severity of symptoms**			X	X	X	X	X	X
**Health services use**				X	X	X	X	X
**Mortality**				X	X	X	X	X
**Sick leave days**							X	
**Side effects and concomitant medication**				X	X	X	X	X
**Treatment compliance**				X	X	X	X	

*Phone call visits

#### Inclusion visit (day 1)

Investigators will collect the following baseline information: sociodemographic data, clinical data, use of concomitant medication, health-related quality of life associated with respiratory symptoms, functional status post-COVID-19, and scoring of asthenia, headache, mental confusion, ageusia, and anosmia.

Participants will perform the 1MSTS test to evaluate the degree of dyspnea on exertion and oxygen desaturation.

A baseline blood test will be performed, including complete blood count, electrolytes, kidney function, liver function, C-reactive protein, creatine kinase, ferritin, d-dimer, type B natriuretic peptide, antinuclear antibodies, lupus anticoagulant, and quantitative anti-SARS-CoV-2 antibodies.

Patients will receive a bottle with the medication (montelukast or placebo), and an appointment for the next visit (on day 7) will be scheduled.

The patient will be asked to return the bottle with the study medication at the end of the study.

#### Office visit (days 14 and 28)

Evaluation of symptoms, treatment adherence, 1MSTS test and oxygen desaturation, and hospitalization since the previous visit will be assessed, as well as the use of concomitant medication, evaluation of adverse events, and compliance with study medication.

#### Phone call (days 7, 21, and 56)

Health-related quality of life associated with respiratory symptoms, post-COVID-19 functional impairment, and symptom progression will be evaluated by means of a telephone call using the same questionnaires. Information regarding the number of visits to the health center, primary and/or hospital emergency care, and hospitalization since the previous visit will be assessed, as well as the use of concomitant medication, evaluation of adverse events, and compliance with study medication.

#### Visit 5 (day 28)

Visit 5 is the last office visit and end of treatment. Patients will be asked to return the bottle with the study medication, and treatment compliance will be evaluated. We will administer the same questionnaires and collect the same data of previous visits.

Relevant concomitant care and interventions are permitted during the trial, with the exception of antileukotriene use.

### Data collection, data management, and quality assurance procedures

Study data will be collected and managed using REDCap, hosted at the Institut Universitari de Recerca en Atenció Primària Jordi Gol i Gurina (IDIAPJGol). REDCap (Research Electronic Data Capture) is a secure, web-based software platform designed to support data capture for research studies, providing (1) an interface for validated data capture, (2) audit trails for tracking data manipulation and export procedures, (3) automated export procedures to common statistical packages, and (4) procedures for data integration and interoperability with external sources [36, 37].

Only the principal investigator or those who have permission can access the data.

A risk approach monitoring plan will be developed and followed via periodic on-site/online visits. Investigators will be instructed in Good Clinical Practice and specific standard operational procedures for the trial.

### Statistical analysis

Baseline characteristics of the sample will be described for each group using mean and standard deviation for quantitative variables and absolute and relative frequencies for qualitative variables. Bivariate comparison of characteristics between patients taking montelukast and patients taking placebo will be carried out to verify group comparability. The Wilcoxon test will be used for the comparison of quantitative variables and the chi-square test for the comparison of qualitative variables (or the Fisher test in case of extreme distributions in the crossed tables). In all comparisons, the statistical significance will be set at 5%.

Outcome measures will be described and compared between the montelukast and placebo groups using the same statistics. No multivariate analysis will be performed.

For the primary endpoint analysis (montelukast efficacy) and secondary endpoints, we will primarily use an intention-to-treat analysis. We will also conduct a per-protocol analysis. R-4.0.2 for Windows will be used.

Additional subgroup analysis will be performed based on symptom duration (< 6 months and ≥ 6 months).

## Discussion

The main goal of this study is to demonstrate the efficacy of montelukast, a previously approved and commercialized drug, in reducing dyspnea and other persistent symptoms in patients with long COVID.

Since it is a new condition, there are no validated scales that evaluate long COVID symptoms and quality of life. The mMRC scale is a self-rating tool validated for COPD and interstitial lung diseases to measure the degree of disability caused by dyspnea on daily activities. The main variable of the study is quality of life as measured by the CAT scale. CAT is a short, simple, standardized assessment test completed by patients to measure health-related quality of life in patients with COPD, providing a reliable and standardized measurement of health [29, 30] This scale was chosen because the validated quality of life scales for long COVID are not yet available. Both scales were selected to cover a wide range of disease severity, with the intention that the greatest discriminating power would be in the mild to moderate range. We will also use the 1MSTS test, a clinical scale to assess dyspnea during exercise. The 1MSTS test has been validated for use in COPD and pulmonary fibrosis and has been recommended in long COVID [31]. The recently developed Post-COVID-19 Functional Status Scale has also been included in the evaluation [32].

Different publications have lately proposed the use of montelukast in the acute phase of COVID-19 because of possible antiviral and anti-inflammatory effects [22–25]. It has been postulated that due to the affinity of montelukast for the ACE receptor, it could interfere with the entry of SARS-CoV-2 into the cell. Consequently, montelukast could shorten the course and severity of acute COVID-19 [23]. Additionally, it has been hypothesized that montelukast could reduce the replication cycle of the virus [24].

Clinical experience has suggested that montelukast may reduce clinical deterioration among hospitalized COVID-19 patients [25].

Two previously registered clinical trials will evaluate the efficacy of montelukast during acute SARS-CoV-2 infection in non-hospitalized patients [26, 27]. One trial aims to evaluate the efficacy of montelukast in reducing the number of emergency room visits and hospital admissions [26]. The second trial will evaluate the efficacy of montelukast and favicovir in decreasing the number of hospital admissions [27].

No treatments have been evaluated for long COVID. The severity of the COVID-19 pandemic with its huge human and economic cost, together with the anticipated long COVID wave, demands urgent, effective therapies to reduce complications associated with the SARS-CoV-2 infection. Based on previous clinical experience [28], we expect to generate evidence on the effect of montelukast in improving quality of life related to respiratory symptoms in patients with long COVID.

## Ethics and dissemination

This study was approved by the Clinical Research Ethics Committee of the IDIAPJGol (reference number 21/091-C) and authorized by the Spanish Agency of Medicines and Medical Products (AEMPS, EudraCT number 2021-000605-24). It has been considered a low intervention trial, following European legislation in Clinical Trials. It has been registered at ClinicalTrials.gov (NCT04695704). This study protocol has been written in accordance with the Standards Protocol Items: Recommendations for Interventional Trials (SPIRIT). Relevant amendments will be submitted to the appropriate authorities (ethics committee and/or AEMPS) for approval. Investigators and patients will be properly informed about the protocol changes. Patients will be re-consented if needed. Changes will be communicated to the study registries.

The clinical trial will be conducted in accordance with the protocol and the Tripartite Harmonized Guide (ICH) for Good Clinical Practice (GCP). The authors guarantee compliance with the General Regulation of Data Protection (RGPD) EU 2016/679, approved by the European Parliament on April 27, 2016, and the tenets of the Declaration of Helsinki and the Belmont Report.

Participants will be properly informed of the clinical trial, have enough time to decide and sign the informed consent form prior to the start of the trial, and their participation will be reflected in their medical records. Participant specimens can only be used for the purpose described in the protocol and informed consent, and they will be destroyed by the end of the study.

Participants’ data will be collected using the REDCAP platform hosted by the IDIAPJGol servers. The data will be stored in the local web server, only accessible to computers with a trusted VPN connection and secure credentials. Only the application service can send the data to the back office, with a firewall that only allows requests from the application IPs. The web server enables you to configure the HTTP X-Frame-Options caption with the value “same-origin” to prevent clickjacking attacks. The final trial dataset will be only accessible for the data management investigators of the research team.

The results of the study will be published in open access, peer-reviewed journals. To ensure quick translation of the research findings into clinical practice, we will conduct webinars on the management of long COVID in primary care, to increase awareness and understanding about long COVID among primary health professionals.

The authorship eligibility will be based on the author’s contribution.

## Data Availability

The datasets generated during and/or analyzed during the current study are not publicly available due to data confidentiality but are available from the corresponding author on reasonable request. Access to data: The data that support the findings of this study is available on request from the corresponding author (Francisco Mera Cordero, fmera@ambitcp.catsalut.net) The data are not publicly available due to the ethical restrictions and confidentiality of research participants.

## Abbreviations

AA: Adverse event
SAE: Serious adverse event
AR: Adverse reaction
SUAR: Severe and unexpected adverse reaction
AEMPS: Spanish Agency for Medicines and Health Products
GCP: Good Clinical Practice
mERC: Ethical Committee for Research with Medicines
DCN: Data Collection Notebook
eDCN: Electronic Data Collection Notebook
ICH: International Conference on Harmonization
ICS: Catalan Institute of Health
IDIAPJGol: University Institute for Primary Health Care Research Jordi Gol i Gurina
CAT: COPD Assessment Test Scale
mMRC: Modified Medical Research Council Scale
COPD: Chronic obstructive pulmonary disease
1MSTS: 1-min sit-to-stand test
PCFS: Post-COVID Functional Status Scale
