# Correction to: Double-blind placebo-controlled randomized clinical trial to assess the efficacy of montelukast in mild to moderate respiratory symptoms of patients with long COVID: E-SPERANZA COVID Project study protocol

**DOI:** 10.1186/s13063-022-06073-7

**Published:** 2022-02-09

**Authors:** Francisco Mera-Cordero, Sara Bonet-Monne, Jesús Almeda-Ortega, Ana García-Sangenís, Oriol Cunillera-Puèrtolas, Sara Contreras-Martos, Gemma Alvarez-Muñoz, Ramon Monfà, Marina Balanzó-Joué, Rosa Morros, Betlem Salvador-Gonzalez

**Affiliations:** 1 Primary Care EAP El Pla Sant Feliu de Llobregat, Primary Care Management Costa de Ponent, Catalan Institute of Health, L’Hospitalet de Llobregat, Spain; 2 Institut Universitari de Recerca en Atenció Primària Jordi Gol i Gurina (IDIA PJGol), Barcelona, Spain; 3 Pharmacy Unit SAP Baix Llobregat Centre, Primary Care Management Costa Ponent. Catalan Institute of Health, Cornellà de Llobregat, Spain; 4 Primary Care Research Support Unit (USR) Costa de Ponent, Primary Care Management Costa de Ponent, Catalan Institute of Health, Cornellà de Llobregat, Spain; 5 Clinical Trials Research Unit (UICEC) IDIAPJGol, Platform SCReN, Barcelona, Spain; 6 Primary Care Research Support Unit (USR) Costa Ponent, Institut Universitari de Recerca en Atenció Primària Jordi Gol i Gurina (IDIAPJGol), Cornellà de Llobregat, Spain; 7 Primary Care Covid Notification and Monitoring Unit UNSC Metropolitana SUD, Primary Care Management Costa de Ponent, Catalan Institute of Health, L’Hospitalet de Llobregat, Spain; 8 Department of Pharmacology and Therapeutics, Universitat Autònoma de Barcelona, Bellaterra, Spain

**Correction to: Trials 23, 19 (2022)**

**https://doi.org/10.1186/s13063-021-05951-w**

Following the publication of the original article [1], we were notified that the author names have been incorrectly tagged (part of the last names had originally been tagged as first name).

- Originally published author names: Francisco Mera Cordero, Sara Bonet Monne, Jesús Almeda Ortega, Ana García-Sangenís, Oriol Cunillera Puèrtolas, Sara Contreras-Martos, Gemma Alvarez Muñoz, Ramon Monfà Escolà, Marina Balanzó Joué, Rosa Morros Pedrós and Betlem Salvador-Gonzalez
- Corrected author names: Francisco Mera-Cordero, Sara Bonet-Monne, Jesús Almeda-Ortega, Ana García-Sangenís, Oriol Cunillera-Puèrtolas, Sara Contreras-Martos, Gemma Alvarez-Muñoz, Ramon Monfa, Marina Balanzo-Joué, Rosa Morros, Betlem Salvador-Gonzalez

The original article has been corrected.
